# Medium Access Control for Opportunistic Concurrent Transmissions under Shadowing Channels

**DOI:** 10.3390/s90604824

**Published:** 2009-06-18

**Authors:** In Keun Son, Shiwen Mao, Seung Min Hur

**Affiliations:** 1 Department of Electrical and Computer Engineering, Auburn University, Auburn, AL 36849, USA; E-Mail: izs0001@auburn.edu; 2 Center for u-Manufacturing, Pohang University of Science and Technology, Pohang, Korea; E-Mail: hsm@postech.ac.kr

**Keywords:** exposed terminal, IEEE 802.11 MAC, Log-normal shadowing, media access control, multi-hop wireless networks

## Abstract

We study the problem of how to alleviate the exposed terminal effect in multi-hop wireless networks in the presence of log-normal shadowing channels. Assuming node location information, we propose an extension of the IEEE 802.11 MAC protocol that sched-ules concurrent transmissions in the presence of log-normal shadowing, thus mitigating the exposed terminal problem and improving network throughput and delay performance. We observe considerable improvements in throughput and delay achieved over the IEEE 802.11 MAC under various network topologies and channel conditions in ns-2 simulations, which justify the importance of considering channel randomness in MAC protocol design for multi-hop wireless networks.

## 1. Introduction

A Media Access Control (MAC) protocol is designed for coordinating access to shared channel(s) among multiple users in order to avoid collisions and achieve efficient use of the medium. It has been shown that the IEEE 802.11 MAC [1], although widely adopted, suffers low throughput performance in the multi-hop wireless network environment [2–4]. In the IEEE 802.11 MAC, the nodes around a transmitter and the target receiver are regarded as potentially interfering nodes. The virtual carrier sensing mechanism is used to prevent these nodes from initiating their transmissions. However, there are scenarios that some of the neighboring nodes' transmissions will not cause collision with, and will not be interfered by the ongoing transmission, but are still forbidden to transmit. Such nodes are termed “exposed terminals” and in such situations the channel spectrum is not efficiently utilized. It has attracted considerable interest to solve or alleviate the exposed terminal problem, since the IEEE 802.11 MAC is becoming the most popular MAC protocol for single- and multi-hop wireless networks.

There have been considerable research efforts on this aspect. For example, MAC protocols requiring additional hardware or PHY capacities are shown to be helpful [5, 6]. In addition, there have been proposals on tuning the carrier sensing range [7–9], controlling the transmit power [10–12], and modifying the behavior of the IEEE 802.11 MAC [13–15]. However, these studies are conducted assuming deterministic wireless propagation models, such as the free-space propagation model or the two-ray ground reflection model [16]. In these models, path loss is determined by the distance between the transmitter and receiver deterministically. However, due to obstacles, multi-path propagation, and mobility, randomness such as shadowing or fading exists in most wireless networks and should be considered in MAC protocol design.

In a wireless network environment, factors such as reflection, diffraction, and scattering affect the propagation of radio waves. In addition to power attenuation, “large-scale shadowing” and “small-scale fading” are usually experienced by radio signals. Small-scale fading describes the rapid fluctuation of the signals over a short period of time or distance. On the other hand, large-scale shadowing represents a random effect which occurs over a large number of measurement locations which have the same distance between the transmitter and the receiver, but have different levels of obstacles on the propagation path. It is well-known that the log-normal shadowing propagation model captures this effect. The log-normal shadowing propagation model describes the random variation of the received power around the mean (nominal) value, and the power variation in decibel (dB) follows a normal distribution [16].

**Figure 1.**
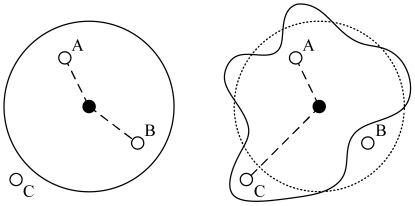
Transmission ranges for the two-ray ground reflection model (left) and the shadowing model (right).

Figure 1 illustrates the transmission ranges when the two-ray ground reflection model (left) and the log-normal shadowing propagation model (right) are used. Under the deterministic channel model, transmission range of a node is circular for a given transmit power, as shown in the left figure in Figure 1. Under shadowing channels as in a typical wireless network environment, the transmission range is not circular anymore. As can be observed in the right figure, although Node *B* is within the mean transmission range of the center node (the dotted circle), it may not receive the center node's transmission due to shadowing. On the other hand, although Node *C* is out of the mean transmission range of the center node, it can receive the center node's transmission. The free space or two-ray ground reflection channels do not model the actual radio propagation precisely, and such inaccuracy may have a considerable impact on the MAC protocol performance since the set of one-hop neighbors is not deterministic anymore. Such randomness caused by shadowing effects should be taken into account in the MAC protocol design to avoid potential collisions and leverage spacial reuse.

Motivated by these observations, in this paper we study the problem of how to mitigate the exposed terminal problem in the presence of log-normal shadowing channels. We propose a location-assisted extension to the IEEE 802.11 MAC protocol for opportunistically scheduling “concurrent” transmissions in the neighborhood of a “free” transmission, i.e., the transmission between the two nodes that first win the channel with RTS/CTS handshake. We assume node location information as in many prior works (e.g., the class of geographic routing protocols [17, 18]). We assume that such location information can be obtained via the global positioning system (GPS) if such service is available, or by using an effective localization scheme proposed in the literature [19]. However, our main objective is to exploit location information for improved network-wide performance, while localization is not the focus of this paper.

We first derive an analysis on the success probability of concurrent transmissions from exposed nodes, which depends on i) the distance between the tagged transmitter and receiver, ii) the distance between the receiver and other interfering nodes, iii) the path-loss exponent, and iv) the parameters of log-normal shadowing. If the computed success probability is larger than a prescribed threshold, concurrent transmissions of exposed nodes are allowed by the proposed scheme. Furthermore, we develop an extension to the IEEE 802.11 MAC to incorporate the above analysis for validating the feasibility of a concurrent transmission, and for scheduling feasible concurrent transmissions. We also describe a simple scheme to estimate the channel parameters if they are not known a priori or if the channels are not stationary. Finally, we implement the proposed location-assisted MAC protocol in ns-2 and compare its performance with the original IEEE 802.11 MAC with extensive simulation studies. We observe considerable gains in throughput and delay achieved by the proposed MAC protocol over IEEE 802.11 MAC, which not only demonstrate the efficacy of the proposed scheme, but also justify the importance of considering channel randomness in MAC protocol design.

The remainder of this paper is organized as follows. In Section 2, we review related work on improving the IEEE 802.11 MAC performance. In Section 3, we discuss the shadowing channel model and success probability of a concurrent transmission. We present the location-assisted MAC extension in Section 4 and evaluate its performance with extensive ns-2 simulations in Section 5. Section 6 concludes this paper.

## 2. Related Work

The IEEE 802.11 MAC protocol is widely adopted in various wireless networks. Although the hidden-terminal problem is effectively solved by the virtual carrier sensing mechanism, the exposed-terminal problem still exists, causing reduced utilization of wireless medium. There have been considerable prior work on improving the spatial reuse of IEEE 802.11 MAC. For example, there are schemes focused on analyzing and adjusting the carrier sensing range [7–9, 20, 21] and control the transmission power [10–12, 22, 23]. Some researchers tried to modify the behavior of current IEEE 802.11 MAC protocol [13, 24] or the physical layer [14]. Some MAC protocols took advantage of additional hardware devices or advanced physical layer technologies such as an additional transceiver, multiple-input and multiple-output (MIMO), and directional antennas [5, 6, 25, 26].

To improve spatial reuse, Ye, Yi and Sikdar [20] proposed a scheme called Aggressive Virtual Carrier Sensing (AVCS) for activating idle nodes within a reserved range. The basic idea is that any node that receives RTS or CTS packet but not both considers the channel is idle and is free to send. The AVCS scheme may cause additional collisions since the exposed nodes do not consider status and location of their target receiver. Note that the use of directional antennas can also improve spatial reuse, since the area consumed by a transmission is reduced due to the more focused transmissions [5, 6, 26]. However, scheduling of node transmissions become non-trivial since one needs to coordinate the directions of the transmitting antenna and receiving antenna in order to establish a link.

Some researchers show that manipulating carrier sensing range can be helpful. Zhu, *et al.* [21] proposed to tune the physical carrier sensing threshold to enlarge the sensing range, such that the entire interference area is covered. With properly tuned physical carrier sensing threshold, all potential interfering nodes will be eliminated, and there is no need for the RTS/CTS handshake. Power control can be used to reduce the interference area such that more concurrent transmissions can be allowed. In [12], Zhou and Nettles suggested a power control scheme that can eliminate hidden nodes and limit exposed nodes by balancing the carrier sensing range and interference range. The achieved performance improvements, however, are modest as observed in the simulation results presented in this paper.

In the MACA-P scheme, a control gap is introduced between the RTS-CTS exchange and the subsequent DATA-ACK exchange, which is used for exposed nodes to transmit their RTS-CTS and for the alignment of the scheduled DATA frame transmission with the current DATA frame transmission [13]. Although some improvements in throughput is achieved in simulations, the additional fixed control gap increases the overall control overhead of IEEE 802.11-like MACs. Such overhead was shown to be as high as 40% in a sensor platform [27], another limiting factor on the IEEE 802.11 MAC performance [4].

In [28], Shukla, Chandran-Wadia, and Iyer presented a simple scheme to enable nodes to identify themselves as exposed terminals and opportunistically schedule transmission of a small frame without RTS-CTS exchange. Note that this scheme does not verify the feasibility of scheduled transmissions and the case of multiple scheduled transmissions is not considered, which may cause collision among themselves and high cumulative interference at the current receiver. Simulation results in [28] showed small throughput improvement when the number of nodes is large.

The idea of location assisted MAC for concurrent transmissions was exploited in our prior work [15], which considers the two-ray ground propagation model. This work is an extension of the scheme in [15] by considering wireless channel dynamics, i.e., shadowing channels. Although shadowing channels were considered in several studies of the connectivity problem of wireless networks [29, 30], most existing MAC schemes adopt the deterministic channel model [15, 31]. Due to channel dynamics in typical wireless environments, the neighbors of a node are not deterministic anymore, which would have considerable impact on MAC protocol design and performance. We propose an opportunistic MAC considering shadowing channels and demonstrate its performance with ns-2 simulations. The performance gain over the IEEE 802.11 MAC is largely due to the consideration of channel dynamics in the MAC design.

## 3. Success Probability under Shadowing Channels

### 3.1. Log-normal Shadowing Channel Model

The free space and two-ray ground reflection models were widely used in prior work. According to such channel models, the transmission range of a node is circular, and all of the nodes within the disk are one-hop neighbors. In typical wireless network environments, however, the variations in the received powers as measured by different receivers with the same distance from a same transmitter are random and independent [32], and can be characterized by the log-normal slow fading [16]. Consider a pair of transmitter and receiver where the distance between them is *d*. According to the log-normal shadowing propagation model, the received power of the intended signal at the receiver in dB, denoted as *P_r,dB_*, is represented by [16]:

(1) $$P_{r,dB}=P_{0,dB}-10\beta \log\left(\frac{d}{d_{0}}\right)+X_{r}$$

where *P*_0_,*_dB_* is the reference power measured at a distance of *d*_0_ in dB, *d*_0_ is the reference distance, *β* is the path-loss exponent, and *X_r_* represents a *normal* random variable with zero mean and standard deviation *σ_dB_*. That is, *X_r_* is a random variable with the following normal probability density function,

(2) $$f_{X_{r}}(x)=\frac{1}{\sqrt{2\pi }\sigma_{dB}}\exp\left(-\frac{x^{2}}{2\sigma_{dB}^{2}}\right)$$

In (1), the received power consists of two parts: the deterministic attenuation depending on the distance between the transmitter and the receiver, and the probabilistic part *X_r_*. The variation of the received power is determined by the standard deviation *σ_dB_*. Note that typically *σ_dB_* ranges from 4 dB to 12 dB in outdoor environments [33]. Due to the probabilistic part *X_r_*, the transmission range of a sending node is not circular anymore, as shown in Figure 1. The received power can be expressed in unit of Watts as follows:

(3) $$P_{r}=P_{0}10^{-\beta \log\left(\frac{d}{d_{0}}\right)+0.1X_{r}}=C\left(\frac{Y_{r}}{d^{\beta }}\right)$$

where *P*_0_ is the reference power measured at *d*_0_ in unit of Watts, $C=P_{0}d_{0}^{\beta }$ is a constant, and *Y_r_ =* 10^(0.1^ *^X^_r_*^)^ represents a log-normal random variable with zero mean and standard deviation $\sigma =\left(\frac{\ln10}{10}\right)\sigma_{dB}$.

### 3.2. Success Probability of the Concurrent Transmission

We next derive the success probability of a transmission in the presence of other interfering transmissions. Suppose that there are *N* interfering transmitters around a tagged receiver, and that the distance between interfering Node *i* and the receiver is *r_i_*. The distance between the tagged transmitter and receiver is *d*. Similar to (3), the interference by interfering Node *i, P_i_*, is represented by:

(4) $$P_{i}=P_{0}10^{-\beta \log\left(\frac{r_{i}}{d_{0}}\right)+0.1X_{i}}=C\left(\frac{Y_{i}}{r_{i}^{\beta }}\right)$$

where *Y_i_* is a log-normal random variable with zero mean and standard deviation *σ*. From (4), the cumulative interference *P_I_* measured at the receiver amounts to:

(5) $$P_{I}=\sum_{i=1}^{N}P_{i}=\sum_{i=1}^{N}C\left(\frac{Y_{i}}{r_{i}^{\beta }}\right)$$

From (3) and (5), we obtain the signal-to-interference ratio (SIR) at the tagged receiver as:

(6) $$\frac{1}{\text{SIR}}=\frac{P_{I}}{P_{r}}=\frac{\sum_{i=1}^{N}C\left(\frac{Y_{i}}{r_{i}^{\beta }}\right)}{C\left(\frac{Y_{r}}{d^{\beta }}\right)}=\sum_{i=1}^{N}{\left(\frac{d}{r_{i}}\right)}^{\beta }\left(\frac{Y_{i}}{Y_{r}}\right)$$

In order to receive and decode the packet successfully, the SIR at the tagged receiver should be greater than a threshold ***T****_SIR_*. The probability of such an event is:

(7) $$P_{\text{succ}}=\mathrm{Pr}\{\text{SIR}>T_{\text{SIR}}\}=\mathrm{Pr}\left\{\frac{1}{Y_{r}}\sum_{i=1}^{N}{\left(\frac{d}{r_{i}}\right)}^{\beta }Y_{i}<\frac{1}{T_{\text{SIR}}}\right\}$$

Let *U_t_ = (d/r_s_*)*^β^ Y_i_*, which is a log-normal random variable with mean *μ_i_* **=** *β* ln (*d/r_s_*) and variance

*σ*^2^. Furthermore, let $V=\sum_{i=1}^{N}U_{i}$. Since *U_i_*'s are independent, *V* can be approximated by a log-normal random variable *W* with the following mean *μ_w_* and variance $\sigma_{w}^{2}$ according to the Fenton-Wilkinson approximation method [34]:

(8) $$\left\{ \begin{array}{l} \mu_{w}=\log(\sum_{i=1}^{N}e^{\mu_{i}})+\sigma^{2}-\frac{\sigma_{w}^{2}}{2}\\ \sigma_{w}^{2}=\log\left[(e^{2\sigma^{2}-1})\frac{\sum_{i=1}^{N}e^{2\mu_{i}}}{{\left(\sum_{i=1}^{N}e^{\mu_{i}}\right)}^{2}}+1\right] \end{array}\right.$$

Finally, (*W/Y_r_*) is a log-normal random variable with mean *μ_w_* and variance $\left(\sigma_{w}^{2}+\sigma^{2}\right)$.

By utilizing the *logistic distribution* for the cumulative distribution function (CDF) of the log-normal distribution [35], $F(x;\mu ,\sigma )={\left[{\left(\frac{e^{\mu }}{x}\right)}^{\pi /(\sigma \sqrt{3})}+1\right]}^{-1}$, we finally obtain the success probability of the transmission as:

(9) $$P_{\text{succ}}={\left[{\left(T_{\text{SIR}}e^{\mu_{w}}\right)}^{\frac{\pi }{\sqrt{3(\sigma_{w}^{2}+\sigma^{2})}}}+1\right]}^{-1}$$

### 3.3. The Case of a Single Interfering Node

In networks that are not very dense, the number of interfering nodes around a receiver is usually small. Consider the case of a single interfering node, e.g., the one that is the closest to the tagged receiver among all interfering notes. The interfering power of this node may dominate the overall interfering power from all other interfering nodes. Letting the distance between the single interfering transmitter and the target receiver be ***r***, the success probability in (7) is reduced to:

(10) $$P_{\text{succ}}=\mathrm{Pr}\left\{\frac{Y_{i}}{Y_{r}}<\frac{1}{T_{\text{SIR}}}{\left(\frac{r}{d}\right)}^{\beta }\right\}=\mathrm{Pr}\left\{Z<\frac{1}{T_{\text{SIR}}}{\left(\frac{r}{d}\right)}^{\beta }\right\}$$

where *Z = Y_i_/Y_r_* represents a log-normal random variable with zero mean and variance 2*σ*^2^. Applying the logistic distribution, we find the success probability to be:

(11) $$P_{\text{succ}}={\left\{{\left[T_{\text{SIR}}{\left(\frac{d}{r}\right)}^{\beta }\right]}^{\pi /\left(\sigma \sqrt{6}\right)}+1\right\}}^{-1}$$

**Figure 2.**
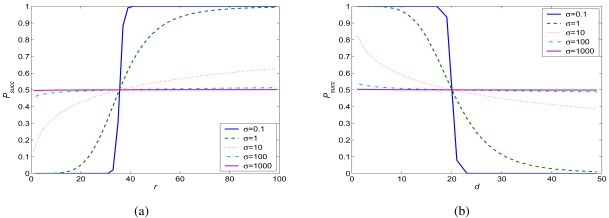
The impact of channel parameters on the success probability *P_succ_* with *T_SIR_ =* 10 and *β =* 4. (a) *P_succ_* vs. *r* when *d =* 20 m. (b) *P_succ_* vs. *d* when *r* = 35.6 m.

As an example, we plot the success probability *P_succ_* for different *σ*'s by evaluating (11) in Figure 2. The parameters are selected as *T_SIR_ =* 10 and *β =* 4 to emulate a typical wireless sensor network scenario. The mean interference range $R_{I}=d\sqrt[{\beta }]{T_{\text{SIR}}}=35.6\text{m}$. We find that *P_succ_* is a decreasing function of *σ* when *r > R_I_*, but an increasing function of *σ* when *r < R_I_*. For example, consider the two extreme cases when *σ =* 0 and *σ =* ∞. When *σ =* 0, the channel is a deterministic one; the received power depends on the distance only and does not have any randomness. Equation (11) becomes a *step function* as: $P_{\text{succ}}=\{\begin{array}{rr} 0, & r<R_{I}\\ 1, & r>R_{I} \end{array}$. That is, there is a perfect channel with the distance is within *R_I_*, and there is no connection atall when the distance is larger than *R_I_*. This is the deterministic disk model used in many prior studies. On the other hand, when *σ* = ∞, the success probability tends to be 1/2 in all cases. It means that the success probability is no longer dependent on the distances *d* and *r*.

## 4. MAC Protocol for Scheduling Concurrent Transmissions

In this section, we describe an extension of the location-assisted MAC protocol presented in our prior work [15], for scheduling concurrent transmissions under log-normal shadowing channels. The key elements of the proposed protocol include identifying an exposed node, validating, and scheduling a concurrent transmission. For completeness, we present the MAC protocol briefly in this section and refer interested readers to [15] for more details. The core module of the extension, the validation of concurrent transmissions, is based on the analysis presented in the previous section. We also show how to estimate the channel parameters when they are not known *a priori*.

### 4.1. Identifying an Exposed Terminal

When a node first overhears an RTS and then a DATA frame from the same transmitter, it can be identified as an exposed terminal with regard to the overheard transmission. In practice, exposed terminals can be identified before receiving the complete frame as follows.

**Figure 3.**
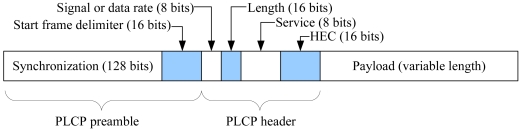
The IEEE 802.11 PHY frame structure when DSSS is used in PHY.

The IEEE 802.11 PHY frame structure when direct spread spectrum sequencing (DSSS) is used is shown in Figure 3. As soon as the physical layer convergence protocol (PLCP) header, which precedes the current MAC DATA frame, is completely received and verified (by checking HEC, a 16-bit CRC code), the exposed node will know the MPDU (MAC protocol data unit) length. The exposed node can determine if the MAC frame is DATA from the length information since DATA frames are always longer than control frames (i.e., RTS, CTS, and ACK). By comparing the Length value with that in the preceding RTS, the exposed node can infer if the RTS and the DATA frame are from the same sender. It can further validate its inference if the interval between the DATA frame and the RTS is (SIFS + CTS + SIFS) plus some propagation delay.

### 4.2. Validating a Scheduled Transmission

Suppose that a node (called “free transmitter”) wins the channel and is transmitting to a one-hop neighbor (called “free receiver”). When a node near the free transmitter identifies itself as an exposed terminal, it can validate the feasibility of its concurrent transmission with a probabilistic approach based on location information and the shadowing channel model. We call such a node a “scheduled transmitter” and its target receiver “scheduled receiver” if the concurrent transmission is successfully scheduled.

When testing the feasibility of concurrent transmission, we focus on the case of two (free and scheduled) transmitters and two (free and scheduled) receivers. This simplification can be justified by the fact that the chance of having multiple interfering transmitters in the neighborhood of a tagged receiver is usually small in many wireless networks. When the path loss exponent is large, usually the interference power from the nearest one dominates the overall interference power. If an identified exposed node has a frame to transmit, it will evaluate the following four probabilities:

- *P_DATA_*_1_ – the success probability of the free DATA frame;
- *P_DATA_*_2_ – the success probability of the scheduled DATA frame;
- *P_ACK_*_1_ – the success probability of the free ACK frame;
- *P_ACK_*_2_ – the success probability of the scheduled ACK frame.

Under log-normal shadowing channels and with location information available, each of the probabilities can be calculated using (11). Table 1 shows the validation procedure validate schdTx(), where *P_th_* is a prescribed threshold. In order to schedule the concurrent transmission, each of the above probabilities should be greater than *P_th_*. As *P_th_* is increased, there will be fewer scheduled transmissions allowed.

### 4.3. The Location-Assisted MAC Protocol

**Figure 4.**
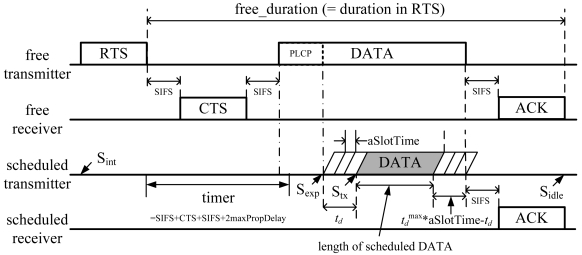
A time-line illustration of the proposed location-assisted MAC protocol.

The operation of the proposed location-assisted MAC protocol is illustrated in Figure 4. First, an exposed node is identified by examining the PHY PLCP frame header, as described in Section 4.1. Once an exposed node is identified, it will try to validate its concurrent transmission, by executing the validation procedure shown in Table 1.

If this test is passed, the exposed node will attempt its scheduled transmission as follows. First, *schdTx_margin* is calculated:

(12) schdTx_margin=(free_duration)−SIFS−CTSSIFS−(PLCP_reading_time)−(schd_data_duration)−SIFS−ACK−(round−trip_prop_delay)

where *free_duration* is the value in the *duration* field of the preceding RTS frame. If *p* = *chdTx_margin* is negative, the scheduled transmission will be canceled. That is, the concurrent DATA transmission cannot be aligned with the free DATA transmission. Otherwise, in order to avoid the case of multiple scheduled transmissions in a small neighborhood, a random delay *t_d_* is introduced as:

(13) $$t_{d}=\left[(\text{rando}m\_\text{integer})\%t_{d}^{\max}\right]\times \text{a SlotTime}$$

where $t_{d}^{\text{max}}=\left\lceil (\text{schdT}x\_m\text{arg}in)/\text{aSlotTime}\right\rceil$.

During the back-off period *t_d_*, the exposed node will keep on detecting if there is a single transmission (i.e., the free transmission) or multiple transmissions (e.g., a scheduled transmission from another exposed node with a smaller *t_d_* value, in addition to the free transmission). Such events can be detected by a sudden increase in received power or bit error rate when decoding the free transmission. If the latter case is detected, the exposed node will not attempt the concurrent transmission; otherwise, it will start sending its DATA frame, which carries the information *T_info_*, defined as:

(14) $$T_{\text{info}}=t_{d}^{\text{max}}-\frac{t_{d}}{\text{aSlotTime}}$$

When a scheduled transmission is received, the scheduled receiver will return an ACK after a delay of *SIFS* + *T_info_* x *aSlotTime*, in order to align its ACK with the ACK from the free transmission.

The proposed scheme assumes location information is available for neighboring nodes. Such location information can be distributed by defining a specific control message. When a node's location is changed, it broadcasts the newly defined control message to all neighbors to update its location information stored at these nodes. Alternatively, we can modify the RTS frame format to piggyback the locations of the sender and its target receiver. When a node overhears an RTS, it extracts the location information, and stores them in a *location table*. Nodes will also exchange their location table with neighbors so that the location of two-hop neighbors are all known after some initialization phase.

### 4.4. Estimation of Channel Parameters

So far we have assumed that the channel parameters, i.e., both *β* and *σ_dB_*, are known. In practice, these parameters may not be given *a priori*. After the initial deployment, the nodes need to measure received signal powers and estimate these parameters, assuming the location information of neighboring nodes are known. We describe how to estimate *β* and *σ_dB_* in the following.

According to the shadowing channel model, the received power *P_r,dB_* is governed by (1), and *X_r_* is normally distributed. Thus, (1) can be categorized as the ANOVA (Analysis of Variance) model I (also known as a fixed-effects model ANOVA) [36]. During network operation, each node computes the distance to other transmitters based on location information, and measures the received powers. Assume that a node has made a set of *n_T_* observations, which consists of *n_i_* observations $P_{r,dB}^{ij}$, *j = 1,…,n_i_*, for each corresponding distance *d_i_,i =* 1, 2, …, *N*. Note that $\sum_{i=1}^{N}n_{i}=n_{T}$. According to the least squares criterion, the sum of the squared deviations of the observations around the expected values, denoted by *Q*, should be minimized:

$$Q=\sum_{i=1}^{N}Q_{i}=\sum_{i=1}^{N}\sum_{j=1}^{n_{i}}{\left(P_{r,dB}^{ij}-\mu_{i}\right)}^{2}$$

where $Q_{i}=\sum_{j=1}^{n_{i}}{\left(P_{r,dB}^{ij}-\mu_{i}\right)}^{2}$.

Differentiating *Q* with respect to *μ_i_*, we obtain that:

$$\frac{dQ}{d\mu_{i}}=\frac{dQ_{i}}{d\mu_{i}}=\sum_{j=1}^{n_{i}}\;\left[-2(P_{r,dB}^{ij}-\mu_{i})\right]$$

Setting the derivatives to zero and replacing *μ_i_* with the least squares estimator *μ̂_i_*, we obtain that:

(15) $${\hat{\mu }}_{i}=\frac{1}{n_{i}}\sum_{j=1}^{n_{i}}P_{r,dB}^{ij}$$

Since μ_i_ = P_0_,_dB_ − 10β log $\left(\frac{d_{i}}{d_{0}}\right)$ according to the attenuation model (i.e, *X_r_* is zero mean), the estimator of the path loss exponent *β*, denoted as *β̂*, can be obtained as follows:

(16) $$\hat{\beta }=\frac{{\hat{\mu }}_{i}-P_{0,dB}}{-10\log\left(\frac{d_{i}}{d_{0}}\right)}=\left(\frac{1}{n_{i}}\right)\sum_{j=1}^{n_{i}}\frac{P_{0,dB}-P_{r,dB}^{ij}}{10\log\left(\frac{d_{i}}{d_{0}}\right)}$$

With (16), we next derive ${\hat{\sigma }}_{dB}^{2}$, the estimator of ${\hat{\sigma }}_{dB}^{2}$. Since the expected value of mean square error (MSE) is equal to ${\hat{\sigma }}_{dB}^{2}$, we derive ${\hat{\sigma }}_{dB}^{2}$ as:

(17) $${\hat{\sigma }}_{dB}^{2}=\text{MSE}=\frac{{\sum_{i=1}^{N}\sum_{j=1}^{n_{i}}\left[P_{r,dB}^{ij}-{\hat{\mu }}_{i}\right]}^{2}}{n_{T}-N}$$

## 5. Simulation Results

In order to evaluate the performance efficiency of the proposed MAC scheme, we implement it using the ns-2 simulator version 2.30 [33] and compare its performance with the original IEEE 802.11 MAC via extensive simulations. We adopt the “Propagation/Shadowing” module among the three ns-2 propagation models in the simulations. The channel and transceiver parameters are chosen to emulate a wireless network with a short transmission range of 26.9 m. For validating a scheduled transmission, *P_th_* is set to 0.5 for the results reported in this section. We choose the shadowing standard deviation of 0.01 dB, for the case of near-deterministic channels, and 4 dB (the default value in ns-2) for the case of moderate shadowing channels. We perform our simulations using the chain, grid, and random topologies as in prior work [14, 21, 25]. The Ad Hoc On Demand Distance Vector (AODV) protocol is used for routing in the simulations [37].

During each simulation, the source nodes start to generate Constant Bit Rate (CBR) traffic at 10 seconds and the simulation terminates after 10 minutes simulation time. The estimation of *β* and described in Section 4.4 is incorporated in the simulations. Through our simulations, we found that the number of failed concurrent transmissions is generally very small for all the case examined, even though the detection mechanism for multiple exposed nodes was not adopted (see Section 4.3). This is due to the fact that the networks have moderate density and the chance of having multiple exposed nodes for a free transmission is relatively negligible. Therefore the detection mechanism is not used for the results presented in this section. For network-wide performance, *goodput*, i.e., the total number of successfully received bytes at the agent level, and end-to-end delay of all received frames are measured for the data sessions. Each simulation is repeated ten times with different random seeds and the average of the ten trials are presented with 95% confidence intervals plotted as error bars in the figures.

### 5.1. Channel Parameter Estimation

We first examine the scheme for estimating channel parameters as described in Section 4.4. During each simulation, the channel parameters *β* and *σ_dB_* are specified in the TCL script file and used in modeling log-normal shadowing channels for each transmission [33]. We assume that the proposed MAC protocol is not aware of the values, and uses the scheme described in Section 4.4 to estimate the parameters based on received signal powers.

We consider a network with a chain topology, where eight nodes are placed in a row with 20 m separation between adjacent nodes. The average transmission range is 26.9 m. The 8-node chain network has two CBR sessions: one from Node 1 to Node 8 with 1,000-byte frames and the other from Node 8 to Node 1 with 700-byte frames. We trace the estimated value of *β* and *σ_dB_* at an intermediate node every 0.01 second for 45 seconds after the sessions are started.

**Figure 5.**
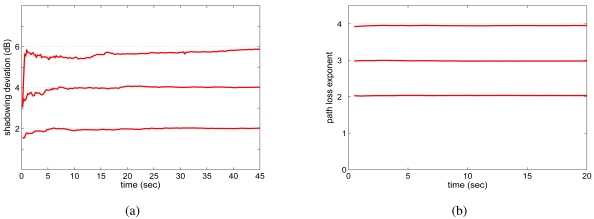
Channel parameter estimation in an eight-node chain network. (a) Estimated channel variance *σ̂_dB_* over time for *β =* 4 and *σ_dB_ =* 2, 4, and 6. (b) Estimated path loss exponent *β̂* over time for *σ_dB_ =* 4 and *β =* 2, 3, 4.

The simulation results are plotted in Figure 5. We observe that in general the estimation schemes are quite effective. The estimated values quickly converge to the actual values set in the TCL scripts. Specifically, the estimation of path exponents *β* is more accurate than that of the standard deviation *σ_dB_* for a given number of samples of received power. Furthermore, the convergence of *β* estimation is much faster than that of *σ_dB_*. The convergence is also slower when *σ_dB_* becomes larger. This is because when the channel exhibits high randomness (as indicated by the larger *σ_dB_* value), more samples are needed to get an accurate estimate. We define the *convergence ratio* for *σ_dB_* estimation as:

(18) $$R_{\sigma }=1-\left|\frac{\sigma_{dB}-{\hat{\sigma }}_{dB}}{\sigma_{dB}}\right|$$

For the worst case when *σ_dB_* = 6, the convergence ratio at 30 seconds is 95.31%. After 45 seconds, the convergence ratio becomes 97.83%. On the other hand, the convergence ratios for the cases of *σ_dB_* = 2 dB and *σ_dB_* = 4 dB become very close to 1 after a few seconds of estimation, due to the lower randomness of the channels.

Similar results are observed when other topologies are used, but are omitted for brevity. The estimation algorithms can be continuously executed due to its low complexity. The estimated values can be updated for every frame received. We conjecture that the proposed approach is also applicable to the case of time-varying channels where the parameters vary over time, as long as they conform to the log-normal slow fading channel model.

### 5.2. Chain Networks

**Figure 7.**
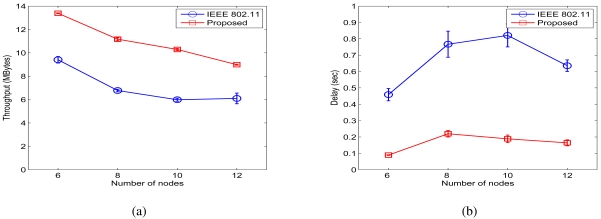
Simulation results for chain networks when *σ_dB_* = 0.01 dB. (a) Throughput. (b) Delay.

We next examine the throughput and delay performance of the proposed scheme using the chain topology, as shown in Figure 7(a). The distance between two adjacent nodes is set to 20 m. The forward flow (from Node 1 to Node *N)* is a CBR session with 1,000-byte packets, while the backward flow (from Node *N* to Node 1) is a CBR session with 700-byte packets. The data rates are set to 90Kb/s, 80Kb/s, 70Kb/s, and 60Kb/s for the 6-, 8-, 10-, and 12-node chain networks, respectively. These data rates are found via extensive simulations, which achieves the maximum throughput the the corresponding chain network. The mean transmission range is equal to 26.9 m, while the mean carrier sensing range is equal to 59.3 m. When the distance between the transmitter and the receiver is 20 m, the mean interference range is computed to be 35.6 m.

Consider first the case that *β =* 4 and *σ_dB_ =* 0.01 dB. In this case, the received power is almost the same as the mean value, with low randomness exhibited. Since the mean transmission range if 26.9 m, a transmitter hardly reaches other nodes except for its two one-hop neighbors. For example, in the case that Node *k* + 1 and Node *k* + 2 concurrently transmit to Nodes *k* and *k* + 3, respectively, the success probabilities of the two transmissions are *P_DATA1_* ≈ 1, *P_DATA_*_2_ ≈ 1, *P_ACK1_* ≈ 1, and *P_ACK2_* ≈ 1. And both transmissions are successful with high probability.

Figure 7 shows the throughput and delay results for the chain network when *σ_dB_ =* 0.01 dB. We observe considerable throughput improvements achieved by the proposed protocol over the IEEE 802.11 MAC. We define the throughput improvement ratio as:

$$R_{\text{thput}}=\frac{\left(\text{thoughput}\_\text{proposed})-(\text{thoughtput}\_820.11\right)}{\left(\text{thoughput}\_802.11\right)}$$

The proposed scheme achieves 42.32%, 64.83%, 71.82%, and 47.32% throughput improvement ratios for the 6-, 8-, 10-, and 12-node chain networks, respectively. In addition, the average end-to-end delay is also drastically reduced for all the cases. We define the delay ratio as:

$$R_{d}=\frac{\left(\text{delay}\_\text{proposed}\right)}{\left(\text{delay}\_802.11\right)}$$

**Figure 8.**
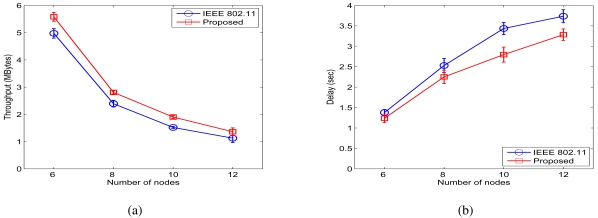
Simulation results for chain networks when *σ_dB_ =* 4 dB. (a) Throughput. (b) Delay.

From Figure 8(b), we find that the proposed scheme achieves delay ratios of 19.49%, 28.63%, 22.98%, and 25.84% for the 6-, 8-, 10-, and 12-node chain networks, respectively. Most concurrent transmissions are successful in this case.

We next consider the case of *β* = 4 and *σ_dB_* = 4 dB. Due to the increased *σ_dB_*, the variation of the received power becomes larger. The one-hop distance may be longer in this case, a phenomenon also observed in the work on connectivity in the presence of log-normal shadowing [29, 30]. In the concurrent transmission scenario, the free and scheduled transmitters are regarded as the interfering node for each other's target receiver. Thus, the possibility of feasible concurrent transmission decreases. For example, when *d =* 20 and *r* = 40, the success probabilities are *P_DATA1_ = P_DATA2_ = P_ACK1_ = P_ACK2_ =* 0.5376. This is smaller than that when *σ_dB_ =* 0.01 dB. Consequently, there are fewer feasible concurrent transmissions when *σ_dB_* gets large.

**Figure 9.**
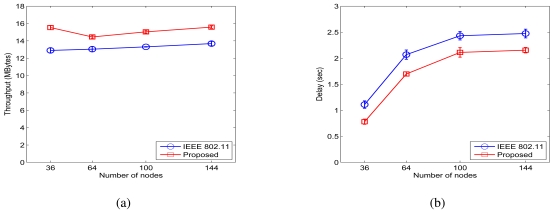
Simulation results for grid networks when *σ_dB_* = 0.01 dB. (a) Throughput. (b) Delay.

Figure 8 shows the simulation results when *σ_dB_ =* 4 dB, where the achieved throughput improvement ratios are 12.21%, 17.27%, 25.10%, and 21.02%, respectively. From Figure 9(b), we find the ratios of the average end-to-end delays are 89.87%, 88.97%, 81.38%, and 87.91%, respectively. Compared to the case of *σ_dB_ =* 0.01 dB, the number of scheduled transmissions are smaller. However, the proposed MAC protocol still achieves considerable improvements in both end-to-end throughput and delay.

### 5.3. Grid Networks

We next examine the performance of the proposed scheme using grid topologies as shown in Figure 7(b). In the grid networks, 36, 64, 100, and 144 nodes are deployed in rows and columns, respectively. The distance from neighbor nodes in the right, left, upper and lower directions is 20 m. As shown in 7(b), half of the border nodes are selected as senders, and the corresponding node on the opposite boarder is the target receiver. A half of the sources transmit a CBR traffic of 1,000-byte frames to the border node on the opposite side, while the rest of sources transmit 700-byte frames.

Throughput and delay results for the case of *σ_dB_* = 0.01 dB are plotted in 9. From the figure, we find that the proposed scheme achieves throughput improvement ratios of 20.40%, 10.77%, 12.98%, and 13.88%, for the 36-, 64-, 100-, and 144-node networks, respectively. In addition, we find from 10(b) that the ratios of the average end-to-end delay with the proposed MAC to that of the IEEE 802.11 MAC are 70.33%, 82.07%, 86.82%, and 88.07%, respectively.

The simulation results for the case of *σ_dB_* = 4 dB for the grid networks are presented in 10. We find that normalized throughput improvements of 22.90%, 24.38%, 38.85%, and 61.74% are achieved. We also find from 11(b) that the ratios of the average end-to-end delay with the proposed MAC to that of the IEEE 802.11 MAC are 85.23%, 91.41%, 87.12%, and 84.36%, respectively. The proposed MAC protocol achieves considerable improvements in both throughput and delay for the grid networks with a regular topology structure.

### 5.4. Random Networks

Finally we study the performance of the proposed protocol under four networks with random topology. For the random networks, 64, 81, 100, and 121 nodes are randomly deployed in a square network area, respectively (see the example in 7(c)). Multi-hop CBR traffic flows are randomly generated with randomly chosen source and destination nodes. A half of the transmitters generate CBR session with 1,000-byte frames, and the rest of them generate CBR sessions with 700-byte frames. All the other configurations of nodes are the same as those in the chain networks.

**Figure 11.**
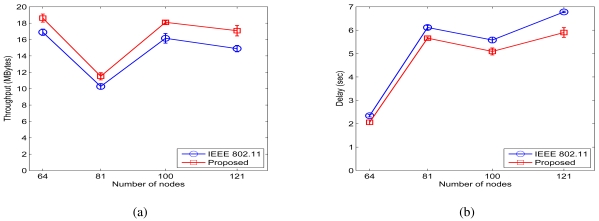
Simulation results for random networks when *σ_dB_* = 0.01 dB. (a) Throughput. (b) Delay.

Figure 11 presents the throughput and delay curves when *β =* 4 and *σ_dB_ =* 0.01 dB, and 12 presents the throughput and delay results when *β =* 4 and *σ_dB_ =* 4 dB. From the two figures, we observe the end-to-end throughput improvement ratios of 10.22%, 12.10%, 12.09%, and 14.72% respectively for the four networks when *σ_dB_ =* 0.01 dB, and throughput improvement ratios of 11.18%, 12.18%, 32.93%, and 28.70% for the case when $\sigma_{dB}^{2}=4\text{dB}$.The proposed scheme also achieves end-to-end delay ratios of 87.96%, 92.56%, 91.25%, and 87.02% when *σ_dB_ =* 0.01 dB and 64.30%, 74.34%, 82.32%, and 83.21% when *σ_dB_* = 4dB.

We also examined the impact of shadowing standard deviation *σ_dB_* on the performance of the proposed scheme. For a 64-node network with a random topology (as shown in 7(c)), we keep all the other parameters intact while increase the standard deviation value from 0.01 dB to 12 dB. Since different network environments have different standard deviation values. Such simulations will help answer the question that under what kind of environments the proposed scheme is effective.

The throughput results are shown in 14(a) and the delay results are shown in 14(b). We find that the normalized throughput improvement ratio ranges from 10.2% to 25.2% when the standard deviation is increased from 0.01 dB to 12 dB. The net improvement, however, is more constant and ranges from 1.6 MB to 2.1 MB. We also observe that the standard deviation has very similar impact on both MAC schemes, as the two curves are roughly parallel to each other. When *σ_dB_* gets larger, the throughputs of both schemes decreases. This is because when the channels become more dynamic, more packets will be lost due to transmission errors, leading to goodput degradation. From 14(b), it can be seen that generally about 0.5 s delay reduction is achieved by the proposed scheme over IEEE 802.11 MAC, except for very large standard deviation values. When standard deviation is 10 and 12 dB, both schemes achieve very small delays. We conjecture that because of the large variation of shadowing channels (and thus the transmission ranges), many packets traverse paths with smaller hop-counts, leading to smaller end-to-end delays for both schemes. Overall the proposed scheme outperforms IEEE 802.11 MAC for all the cases considered in this simulation.

As illustrated in Figure 1, the effect of log-normal fading is two-sided: (i) some nodes (e.g., Node B) that are within the transmission range, are not neighbor anymore; and (ii) some nodes (e.g., Node C) that are not a neighbor but now are neighbors (and thus exposed nodes). For the chain network considered in the simulations, Type (i) effect is more likely to happen, resulting in broken links and lost packets. Therefore the performance improvement when *σ_dB_* = 4 dB is smaller than that when *σ_dB_* = 0.01 dB. When the random topology is used, however, a node has more neighboring nodes within the average transmission range and some other nodes outsider but close to the average transmission range. Both Type (i) and Type (ii) events are equal-likely to happen, and we see similar improvements for both shadowing channels and near-deterministic channels.

There are many factors that affect the performance of a MAC protocol in multi-hop wireless networks, such as topologies, traffic patterns, and channel parameters. Overall our simulation results demonstrate that the proposed location-assisted MAC protocol is effective in achieving considerable throughput and delay improvements over the IEEE 802.11 MAC under various circumstances of log-normal shadowing channels. The improvements clearly justify the need and the benefits of opportunistically scheduling concurrent transmissions while considering log-normal shadowing channels.

## 6. Conclusions

In this paper, we presented a location-assisted MAC protocol under the presence of log-normal shadowing channels. We derived the success probability for the concurrent transmissions from exposed terminals, which is then incorporated into the MAC protocol for validating the feasibility of concurrent transmissions. Estimation techniques are also presented for estimation of channel parameters. Our simulation results showed that the proposed protocol can effectively leverage spatial reuse, and thus improve the end-to-end throughput and delay performance over IEEE 802.11 for various network topologies and channel conditions.

## Figures and Tables

**Figure 6.**
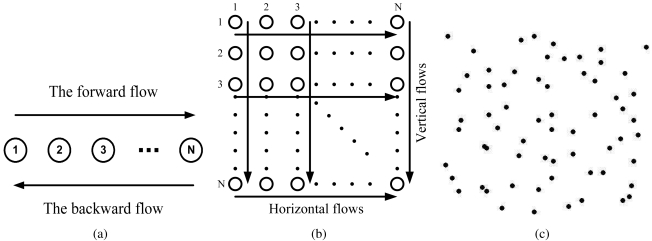
The three types of network topologies used in the ns-2 simulations. (a) Chain topology. (b) Grid topology. (c) Random topology.

**Figure 10.**
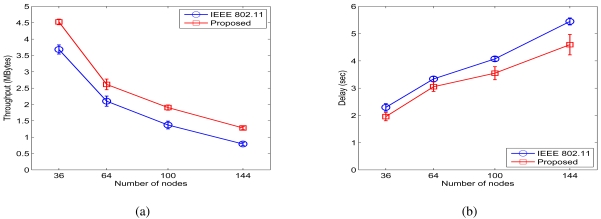
Simulation results for grid networks when *σ_dB_* = 4 dB. (a) Throughput. (b) Delay.

**Figure 12.**
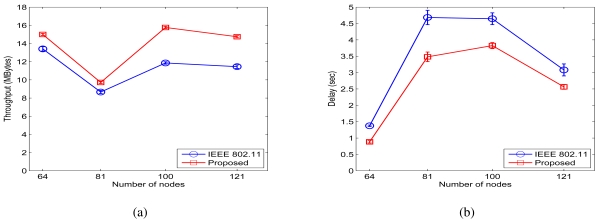
Simulation results for random networks when *σ_dB_ =* 4 dB. (a) Throughput. (b) Delay.

**Figure 13.**
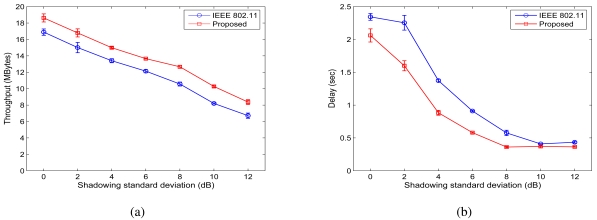
Simulation results for random networks when *σ_dB_* is increased from 0.01 dB to 12 dB. (a) Throughput. (b) Delay.

**Table 1.** The validation procedure for feasibility of a concurrent transmission.

	**Procedure** validate schdTx()
1:	Calculate *P_DATA_*_1_, *P_DATA_*_2_, *P_ACK_*_1_ and *P_ACK_*_2_;
2:	**IF** { (*P_DATA_*_1_*> P_th_*) AND (*P_DATA_*_2_*> P_th_*) AND (*P_ACK_*_1_*> P_th_*) AND (*P_ACK_*_2_*> P_th_*) }
3:	Return 1; // The concurrent transmission is feasible
4:	**ELSE**
5:	Return 0; // The concurrent transmission is not feasible
6:	**ENDIF**
